# Developmental origins of diabetes mellitus: Environmental epigenomics and emerging patterns

**DOI:** 10.1111/1753-0407.13403

**Published:** 2023-05-16

**Authors:** Hong Zhu, Guolian Ding, Xinmei Liu, Hefeng Huang

**Affiliations:** ^1^ Obstetrics and Gynecology Hospital, Institute of Reproduction and Development Fudan University Shanghai China; ^2^ Research Units of Embryo Original Diseases Chinese Academy of Medical Sciences Shanghai China; ^3^ Key Laboratory of Reproductive Genetics (Ministry of Education) Zhejiang University School of Medicine Hangzhou China; ^4^ Shanghai Key Laboratory of Embryo Original Diseases Shanghai China

**Keywords:** diabetes susceptibility, environmental factors, epigenetic modifications, offspring, transgenerational inheritance, 环境因素, 糖尿病易感性, 表观遗传修饰, 子代, 跨代遗传

## Abstract

Mounting epidemiological evidence indicates that environmental exposures in early life have roles in diabetes susceptibility in later life. Additionally, environmentally induced diabetic susceptibility could be transmitted to subsequent generations. Epigenetic modifications provide a potential association with the environmental factors and altered gene expression that might cause disease phenotypes. Here, we bring the increasing evidence that environmental exposures early in development are linked to diabetes through epigenetic modifications. This review first summarizes the epigenetic targets, including metastable epialleles and imprinting genes, by which the environmental factors can modify the epigenome. Then we review the epigenetics changes in response to environmental challenge during critical developmental windows, gametogenesis, embryogenesis, and fetal and postnatal period, with the specific example of diabetic susceptibility. Although the mechanisms are still largely unknown, especially in humans, the new research methods are now gradually available, and the animal models can provide more in‐depth study of mechanisms. These have implications for investigating the link of the phenomena to human diabetes, providing a new perspective on environmentally triggered diabetes risk.

## Graphical abstract

- Interactions of environmental factors and epigenome in early life, via environmentally modified epigenetic markers, affect the transcript profiles of pancreatic islets and peripheral insulin target organs, increasing the susceptibility of developing diabetes.
- Environmentally acquired diabetic phenotype and epigenetic modifications can even be inherited across generations via affecting germline epigenome, providing a new perspective on the alarming increase in diabetes incidence.
- Although there is a long way to applications for clinical practice, “developmental origins of adult diabetes” is an emerging promising perspective for diabetes prevention and intervention.

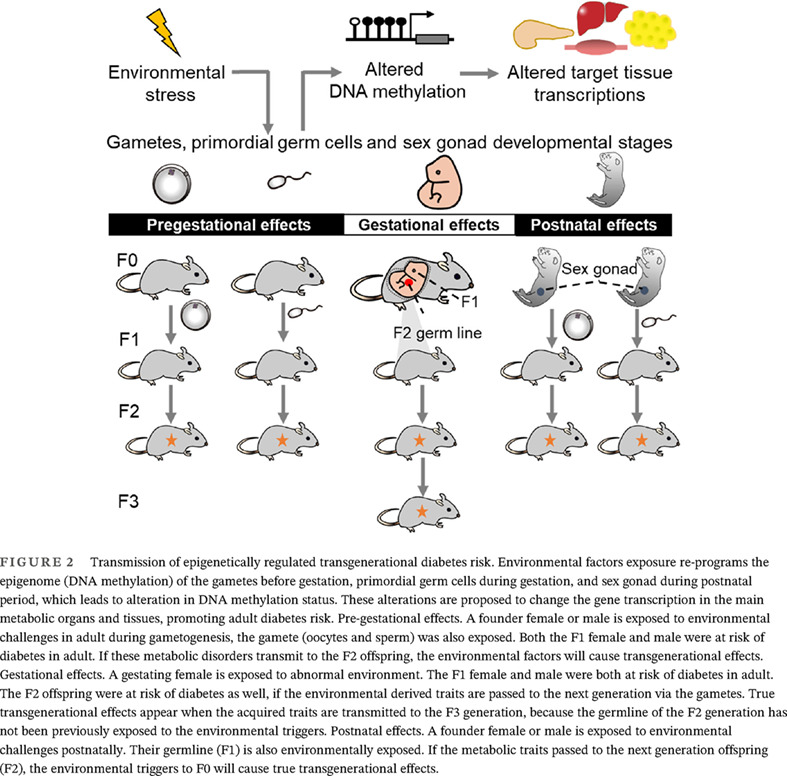

## 摘要

越来越多的流行病学证据表明，生命早期的环境暴露与后期糖尿病易感性有关。此外，环境诱导的糖尿病易感性可遗传给后代。表观遗传修饰提供了与环境因素和可能导致疾病表型的基因表达改变的潜在关联。在本文中，我们梳理了越来越多的证据，表明发育早期的环境暴露与糖尿病通过表观遗传修饰相关。本文首先总结了环境因素通过亚稳态表等位基因和印记基因等表观遗传靶点来修饰表观基因组。然后，以糖尿病易感性为例，概述了表观遗传学在关键发育时间窗、配子发生、胚胎发生、胎儿期和出生后阶段响应环境挑战的变化。虽然其机制仍有很大部分未知，尤其是在人类中，但新的研究方法正在逐步出现，动物模型的策略可以实现对机制的更深入研究。这对研究这些现象与人类糖尿病之间的联系具有意义，为环境触发的糖尿病风险提供了一个新的视角。

The rapid rise in the prevalence of diabetes constitutes a severe threat to individual health and is also a major burden for the global economy.^1^ In the past few decades, diabetes has emerged as a global epidemic: 537 million adults, that is, 1 in 10 (20–79 years) are living with diabetes. This number is predicted to rise to 643 million by 2030 and 783 million by 2045.^1^ Previous research usually examined the diabetes susceptibility based on a combined effect of lifestyle and genetics.^2^, ^3^ One important risk factor is metabolic imbalance, with excess of calorie intake and low energy expenditure.^4^ Another risk factor is individual genetic variation, which highlights the importance of genotype in diabetes development.^5^ However, it appears that an individual's own lifestyle and gene variation are insufficient to explain the alarming increase of diabetes mellitus.^6^

Developmental origins of adult diseases provide a novel view on the diabetes prevalence. A well‐documented case supporting this view is the Dutch Hunger Winter. Individuals exposed to Dutch famine during middle and late gestation were at increased risk for diabetes in adulthood.^7^, ^8^ Thus, the hypothesis that emerged from human epidemiological data proposes that fetal metabolic adjustments to the environment may substantially alter the risk of adult disease predisposition, such as diabetes, cardiovascular disease, and cancer.^9^, ^10^ This thought framework developed into the general concept of “developmental origins of health and disease (DOHaD).”^11^ Nowadays, DOHaD theory has been extended to the “gamete/embryo‐fetal origins of adult disease,” which suggests that gametes and embryos are also susceptible to the environmental factors, and acquired alterations persist despite the lack of sustaining exposure.^12^, ^13^ Further, growing research including human and animal studies supported the developmental origins hypothesis in the field of diabetes, indicating that adverse environmental exposure of gamete, embryonic, or fetal stage may lead to the increased risk of diabetes after birth.^14^

Remarkably, follow‐up on the Dutch Hunger Winter suggested that the exposure of paternal and maternal intrauterine malnutrition increased the risk of obesity in offspring.^15^, ^16^ Evidence from another historical cohort (the Överkalix cohort) also showed that overnutrition in boys could bring an increased risk of diabetes‐related cardiovascular mortality to their grandchildren.^17^, ^18^, ^19^, ^20^ These data indicated that diabetes caused by environmental exposure during early development can additionally transmit the disease susceptibility to the following generation(s). The inheritance of acquired phenotypes by the following generations is referred to as intergenerational or transgenerational effects, which was further supported by the animal studies.^21^, ^22^

The most important question is how transient environmental alterations during early life can lead to long‐term effects. One general mechanism is the changes of epigenetics.^23^, ^24^ “Epigenetics” literally means “above the genetics,” which refers to the stable and mitotically inheritable alterations of gene expression, without changes in the DNA sequence.^25^, ^26^ Obviously, epigenetic modification provide a possible mechanism by which the effects of environmental factors on the epigenome can have long‐term influence on gene expression. Increasing evidence from animal models have indicated that environmental exposure induces a broad range of phenotypes by epigenetically altering gene expression.^27^

Here we focus on the existing evidence and discuss the epigenetic modifications that are likely to be the targets for an increased risk of diabetes due to environmental exposures. Then we discuss the different time windows implicated in developmental origins of diabetes and its epigenetic mechanisms. Finally, we discuss the inter‐ or transgenerational effects of environmentally induced diabetes susceptibility. We conclude the review to determine the importance of environmental epigenetics in human diabetes susceptibility. Such studies might yield novel diabetic prediction, diagnosis, and therapies.

## 1 EPIGENETIC TARGETS OF THE ENVIRONMENTAL FACTORS

**FIGURE 1:**
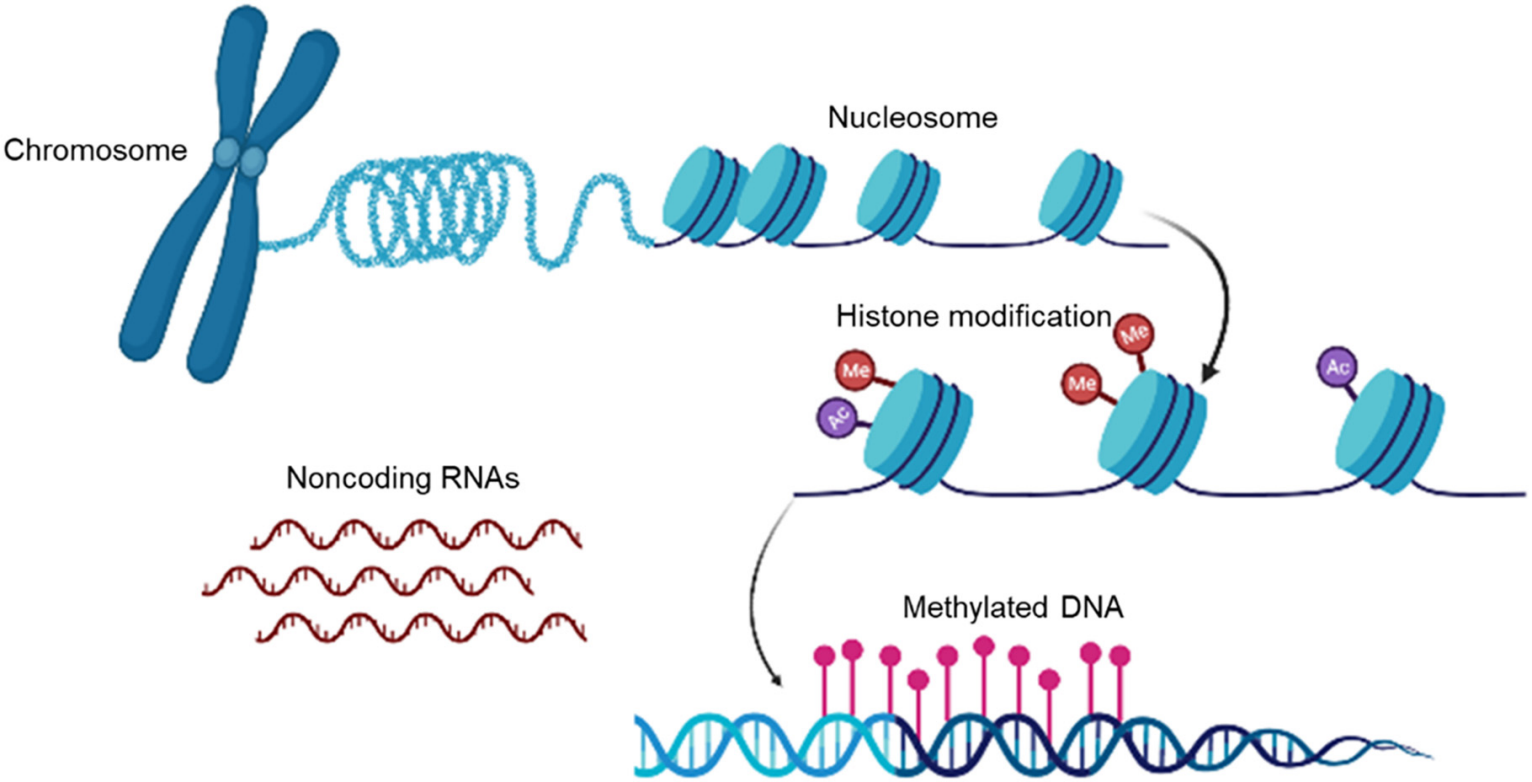
Mechanisms of epigenetics modifications. Gene expression is regulated by genetic as well as epigenetic mechanisms. Shown here are some well‐studied epigenetic mechanisms, including DNA methylation, histone modifications, and the noncoding RNAs‐mediated modulation of gene expression. Some of the mechanisms are passed through cell divisions and contribute to the acquired phenotype.

In many studies, environmentally induced epigenetic modifications are mainly associated with altered DNA methylation or histone modifications^28^, ^29^ (Figure 1). To date, the most studied of epigenetic mechanism is DNA methylation, taking place at the carbon‐5 position of cytosine in cytosine‐guanine (CpG) dinucleotide.^29^ DNA methylation is essential in genomic imprinting and silencing transposable elements.^30^, ^31^ Histone modifications mediate gene expression by changing chromatin packaging of DNA.^32^ Noncoding RNAs, another epigenetic mechanism, may also contribute to the epigenetic regulation, particularly at repeated DNA sequence.^33^ There is evidence that the gene expression, which was regulated by histone, methylation, and noncoding RNAs, can be disturbed by environmental factors.^24^ However, for most histone modifications and noncoding RNAs, it is less clear whether they contribute to environmentally induced traits and inter‐ or transgenerational inheritance.^25^, ^34^

Animal studies have shown that environmental exposures in early life can lead to locus‐specific alterations in the epigenome.^21^ In particular, three genomic targets are vulnerable to the environmental perturbations, including the promoter regions of some housekeeping genes, transposable elements, and regulatory regions of imprinted genes.^24^ These targets regions are rich in CpG dinucleotide sequences, with differential status of methylation or histone modifications in the same region, regulating gene expression.^24^ We mainly focus on the genes with metastable epialleles, imprinted genes, and diabetes‐related functional genes, which have a potential association between the environment during early development and adult diseases susceptibility.

### 1.1 Genes with metastable epialleles

Metastable epialleles are referred to loci that can be epigenetically modified in a variable and reversible manner.^24^ Transposable elements are always silenced by CpG methylation, but there is a subset of transposable elements, whose methylation status is metastable and stochastically alters.^35^ The epigenetic status can influence the adjacent gene expression, which causes epigenetic mosaicism among cells in genetically identical individuals.^35^, ^36^

A classic model is the agouti viable yellow (*A* ^*vy*^) allele in mice. As a metastable epiallele, the loci contain an upstream intracisternal A‐particle (IAP) retrotransposon.^37^ When the retrotransposon is unmethylated, the agouti gene is aberrantly expressed, causing a yellow coat color and diabetes.^38^ Maternal dietary methyl‐donor supplementation could shift the coat color in offspring from yellow to brown, which was associated with an increase in DNA methylation in IAP retrotransposon.^39^ This effect of maternal nutritional environment on the phenotype in offspring was directly linked to the changes in the epigenome.

Methylation profiles at IAP CpG sites are highly correlated in tissues that originated from the three germ layers, suggesting that methylation in response to environmental factors are established earlier than embryonic stem cell differentiation.^40^ These epigenetic alterations are also stable and can readily transmit from one generation to the next via germline.^41^ This phenomenon is the advent of studying the roles of epigenetic modifications during early development in the origins of adult diseases. Human genome is riddled with transposable elements.^42^, ^43^ Therefore, one challenge will be to explore the biological relevance of these changes and whether they affect human diabetes susceptibility.

### 1.2 Imprinted genes

Most autosomal genes are expressed by both paternal and maternal alleles, whereas approximately 1% of the autosomal genes are imprinted, which are expressed from only paternal or maternal allele.^24^, ^44^ Gene imprinting is a non‐Mendelian inheritance pattern, with germline‐inherited and epigenetically regulated features.^45^ Imprinted gene expression is parent‐of‐origin dependent, and imprinted marks are established in parental gametes.^24^ Imprinted gene expression in the individuals also depends on environmental information stored in the previous generation.^24^ On the basic understanding that imprinting alterations can lead to a variety of disease phenotypes, imprinted loci might be the epigenetic targets of the environmental abnormalities that induce the diseases.

Germline or somatic‐cell disorders of imprinting regions or genes in early life has a vital causative role in developmental abnormalities, such as Angelman syndrome and Prader–Willi syndrome.^46^ Recently, numerous research put forth the idea that imprinting genes has been involved in different types of diabetes. Two thirds of transient neonatal diabetes (TNDM) are caused by alterations at chromosome 6q24, where the imprinted genes Plagl1 are located, regulating cell cycle arrest and pancreatic islet insulin secretion.^47^, ^48^ Imprinted genes are also implicated in type1 diabetes (T1D) and type 2 diabetes (T2D). Several identified epigenetically imprinted genes such as *Igf2* (insulin‐like growth factor 2), *Dlk1* (delta‐like 1 homologue), *Kcnq1* (potassium voltage‐gated channel subfamily Q member), and *Cdkn1c* (cyclin‐dependent kinase inhibitor 1C) have been shown to be associated with metabolic syndrome‐related phenotypes and a parent‐of‐origin involved in transgenerational diabetes.^49^, ^50^, ^51^

The parental‐specific regulation of imprinted genes required DNA‐methylation, the process of DNA‐methylation establishment are vulnerable to environmental factors in early life, especially the gametogenesis and early embryogenesis with the genome demethylation and remethylation.^52^ Thus, imprinted genes are potential loci for environmentally induced diseases with parental‐inheritance markers. However, the imprinted genes were found to be strongly associated with species, suggesting that more suitable animal model choice for assessing its function in human phenotype is necessary.

### 1.3 Functional genes with altered epigenetic modification

Maturity‐onset diabetes of the young is a type of early‐onset diabetes that is mostly caused by gene mutations, which encodes transcription factors, such as *IPF1/PDX1*, *HNF1A/4A/1B*, and *NEUROD1*.^2^ One mechanism of *HNF1A* activating gene transcription is chromatin remodeling of promoter regions.^53^ *PDX1* affects glucose‐induced insulin expression in pancreatic islet *β* cells, which requires an interaction between *PDX1* and hyperacetylation of histone H4 at the promoter of insulin gene.^54^, ^55^ TNDM is another type of diabetes that appears in the first 6 weeks of life in growth‐retarded neonates.^47^ Hypomethylation at chromosome 6q24 has been reported in TNDM.^48^ Interestingly, recent studies have shown that *ZFP57* mutations are involved in TNDM and hypomethylation at chromosome 6q24, including the imprinted genes *PLAGL1* and *HYMAI*.^48^ T1D is an autoimmune disease with pancreatic β‐cells destroyed. According to previous studies, T1D‐associated pathogenic genes are the major targets of histone modification. Take *HLA‐DRB1* and *HLA‐DQB1*for example: the acetylation level of H3K9Ac in the upstream regions of *HLA‐DRB1* and *HLA‐DQB1* were increased in patients with T1D, which was further confirmed in in vitro studies.^56^

T2D is a complex type of diabetes caused by a combination of insulin resistance and impaired insulin secretion. Increasing studies have examined the epigenetic alterations in pancreatic islets from patients and animal models. Pancreatic islets of T2D patients showed increased DNA methylation status of the insulin promoter regions in parallel with defective insulin secretion.^57^ Furthermore, DNA methylation sequencing data from the pancreatic islets of T2D and healthy donors have identified a number of epigenetically regulated new target genes that are associated with islet function.^58^ For example, *TCF7L2*, *PPP2R4*, *SLC25A5*, *GRB10*, *CDKN1A*, *PDE7B*, and *SEPT9* were found to be differentially methylation modified and expressed in the T2D patients' pancreatic islets, which impaired insulin and glucagon secretion in β‐cells and α‐cells, respectively.^58^, ^59^

In addition, insulin resistance is also a potential mechanism in an increased risk of T2D, that is a state in which the main target tissues such as skeletal muscle, adipose tissue, and liver, respond inadequately to insulin.^60^ A study suggested that the visceral adipose tissue from insulin‐resistant patients had an altered DNA methylation profile.^61^ Obviously, most of the differentially methylated genes were related to insulin pathway, and 10% of which were identified as genes, for example, *IGF2BP1*, *GATA4*, *TET1*, *ZNF7L4*, and *ADCY9*, associated with diabetes.^60^

## 2 DEVELOPMENTAL STAGE‐DEPENDENT EFFECTS

In mammals, there are two major epigenome reprogramming periods, gametogenesis and embryo‐fetal development.^62^, ^63^ Epigenetic alterations during the process of fertilization, early embryogenesis, and gametogenesis are amplified by cell division and somatic maintenance and, thus, may affect a high proportion of cells in an organ.^64^ Altered epigenetic information that is acquired in early development could be maintained throughout later life at specific genes and some loci. Therefore, the effects of environmental information on the epigenome and diabetes phenotype depend on the developmental period.

### 2.1 Gametogenesis stage

Epigenome is globally reprogrammed at different developmental schedule and magnitude during oogenesis and spermatogenesis.^65^ Erasure of genomic DNA methylation occurs in primordial germ cells and establishment occurs during germline cell differentiation and maturity, which makes germ cells vulnerable to environmental factors, even in adults.^66^ In particular, 90% of histones in male germ cells are replaced by protamines. After fertilization, the paternal and maternal genome are demethylated again and epigenetic marks are reestablished during the first cell division.^62^ The two major rounds of reprogramming events are necessary for the pluripotency and proper embryonic development. Notably, during these processes, any environmentally induced epigenetic modifications in germ cells could probably affect epigenomic reprograming and influence the next generation.

A classic example is the paternal prediabetes model, in which the impaired glucose tolerance and insulin sensitivity was found in offspring, and the transcriptomic and epigenomic profiling of pancreatic islets from offspring exhibited altered gene expression and DNA methylation.^67^ Additionally, paternal prediabetes status changed overall methylome patterns of sperm in F1 offspring, contributing to the transgenerational inheritance.^67^ Recent studies suggest the involvement of tRNAs involved in the paternal intergenerational inheritance.^68^, ^69^ The evidence demonstrated that paternal diet affected the tRNAs in mature sperm, and injection of tRNAs into normal zygotes induced diabetic phenotype and altered gene expression in F1 offspring.^70^ Additionally, a recent study reported that paternal exposure to bisphenol A (BPA, an environment chemical) in rats is associated with impaired glucose tolerance in adult male offspring. These alterations correlated with increased *Igf2* DMR2 methylation in sperm of the F0 and decreased *Igf2* expression F1 pancreatic islets.^71^ This offers novel option for a mechanism of paternal sperm information transmission to the next generation without active transcription and translation.

Similarly, epigenetic marks in maternal germline could also be modified by environmental exposure.^72^ The epigenetic mark of most concern is DNA methylation, which is indeed sensitive to the environmental factors. In mice, maternal high‐fat diet (HFD) and obesity could induce hypermethylation of the *Leptin* promoter and hypomethylation of the *Pparα* promoter.^73^ A previous study has indicated the transmission of diabetic phenotype acquired by maternal HFD via gametes to offspring.^74^ However, epigenetic inheritance via maternal germline remains unclear. Recent research also demonstrated that maternal diabetes transmitted the susceptibility of impaired glucose tolerance to F1 offspring.^75^ This phenomenon occurs by decreased Tet3 level of oocytes, which disturbs paternal genome reprogramming in zygotes, affecting a subset of paternally hypermethylated diabetes‐related gene expression.^75^ This work showed an exciting new explanation for a detail molecular mechanism of how oocyte transmitted information of epigenetic inheritance from the pregestational maternal hyperglycemia environment.

### 2.2 Embryogenesis stage

The early embryonic stage of development appears to be particularly sensitive to environment factors.^76^ In mammals, early embryonic cells contain high levels of epigenetic regulators, including DNA methyltransferases and chromatin regulators.^64^ The plentiful regulatory machineries in early embryos and stem cells are potential reasons for the susceptibility to environmental signals. In contrast, most adult cells are relatively less active, and epigenetic marks have become stabilized.^64^ The potential mechanism can be investigated in animal models and humans, in which assisted reproductive technology (ART) is frequently used.

In rodents, in vitro culture of the zygote and preimplantation embryo was further studied. The culture process itself and the nonphysiological culture micro‐environment may affect postnatal growth and the individual glucose metabolism, including impaired fasting glucose levels and glucose tolerance.^77^ Freezing and thawing of the mouse preimplantation embryos could impair glucose tolerance and hepatic glycogen synthesis in adult offspring, accompanied by the alteration of transcriptomics in hepatocyte.^77^ In human, the effects of ART on a single maternal imprinting‐controlled region have been found, which suggested that 3 of 12 ART‐conceived children showed *KvDMR1* hypomethylation.^78^

Besides ART itself, maternal environment during preimplantation stages can also affect offspring health. ART involves ovarian stimulation, the nonphysiological procedures associated with DNA methylation alterations in blastocysts, which have an intergenerational effect on metabolism in offspring.^79^ In another mouse model, female mice were fed a low protein diet from mating (Embryonic day 0) to implantation (E3.5) stages. During embryogenesis stage, this transient maternal exposure led to an increased adult metabolic disease, with DNA hypomethylation in the adult tissues.^80^ More in‐depth research would shed light on the important role of this critical period for individual metabolic programming and health.

### 2.3 Fetal phase

Many studies have suggested that the fetal stage is susceptible to epigenetic disturbance. The intrauterine developmental period is accompanied with tissue differentiation, organogenesis and fetal growth.^81^ In the *A* ^*vy*^ mouse model, maternal intake of methyl‐donor during pregnancy had significant effects on *A* ^*vy*^ methylation in offspring, but no apparently effect on maternal *A* ^*vy*^ methylation.^64^ A typical study on the Dutch Hunger Winter, indicated that famine exposure in the peri‐conceptional stage caused diabetic phenotype in the next generation.^82^ A minor, but significant alteration in DNA methylation of *Igf2*, *Ins*, and *Gnas* has been reported.^82^, ^83^

Another example of fetal origins of diabetes is the gestational diabetes mellitus (GDM). The evidence from Pima Indians showed that maternal diabetes may increase the risk of T2D in adult life.^84^ The offspring conceived after their mother been diagnosed with diabetic showed a 3.7‐fold higher risk of T2D compared to their siblings born before the mother became diabetic.^84^ Maternal gestational diabetes is associated with DNA methylation variations in placenta and cord blood of the exposed offspring.^85^ 48 differentially methylated CpG sites mapping to 29 genes and 10 intergenic regions were found in Pima Indian offspring exposed to maternal diabetes.^86^ In utero hyperglycemia during the fetal period in mice could also lead to insulin deficiency and increased diabetic risk in adulthood.^87^ This adult phenotype is associated with abnormal *Igf2/H19* DNA methylation modification in offspring pancreatic islets.^87^ Interestingly, maternal insulin therapy of GDM failed to protect offspring when fed with HFD after birth, especially in male offspring, and, DNA methylation profiling of pancreatic islets in offspring also exhibited hypermethylated regions in several genes which regulate insulin secretion, such as *Abcc8*, *Cav1.2* and *Cav2*.^88^

Although altered nutrients during gestational period significantly affects the incidence of diabetes, environmental chemicals exposure also could modify the risk of diabetes. In utero, maternal BPA exposure in rats have been shown to increase glucokinase (*Gck*) promoter methylation, and decrease *Gck* gene expression in the liver of 3‐wk‐old offspring.^89^ Phthalates, another environmental chemical, is also associated with diabetes. Epidemiological data showed that perinatal phthalate exposure is related to abnormal insulin secretion and insulin resistance.^90^ One rat study displayed that perinatal exposure to phthalates increased global DNA methylation and increased histone deacetylase 2 interaction toward *Glut4* in the offspring muscle.^91^

Latest research found that the environmental factors have different effects on the placenta and embryo during gestational stage.^81^ They defined a novel mechanism for intergenerational transmission via placenta. Maternal environmental factors affect fetus by an increased placental superoxide dismutase 3 (SOD3) level through vitamin D receptor, which causes activation of AMPK/TET signaling axis in fetal offspring liver, resulting in DNA demethylation of metabolic genes, altering metabolic phenotype.^92^ Whether above significant differences might be used as potential biomarkers to predict diabetes susceptibility in human, needs future prospects.

### 2.4 Postnatal phase

The postnatal stage is characterized by the metabolic organ maturation, including pancreas, liver, muscle and adipose tissue.^81^ During postnatal stage, environmental effects, especially the nutritional factors, transit from umbilical to oral feeding.^93^ Studies in rodents have shown that in respective cross‐fostering paradigms, pups fed by obese/diabetic dams developed an increased risk in abnormal glucose metabolism in later life.^94^ Additionally, exposures during the lactational stage may amplify the effects of in utero exposures on metabolic disease. For instance, overfeeding during lactation increased adiposity and glucose intolerance in rat offspring from maternal obesity, which provide evidence for the independent contribution of the lactational stage to metabolic syndrome.^95^ In these models, micro‐vesicle RNAs transmission via milk, not epigenetic marks, is more investigated.^81^, ^93^

Environmental factors can affect the postnatal epigenome as well.^64^, ^93^ Data obtained from a monozygotic twins' study demonstrated that locus‐specific inter‐individual DNA methylation differences arise, in part, after birth.^96^ Neonatal feeding of high‐carbohydrate decreased Glut4 transporter mRNA in skeletal muscle in male offspring, accompanied by an increase in *Slc2a4* promoter methylation.^97^ In human, breastfeeding might lead to DNA methylation alteration at *LEPTIN* and *NYP* promoters, which have been reported to be linked to obesity and diabetes.^98^, ^99^ This is in line with the previous animal data, showing that nutrition during this specific stage could affect the individual's epigenome.

Postnatal factor does not only refer to maternal nutritional status but also parental nurturing behaviors. In animal models, maternal care exhibited reduced fear and more modest hypothalamic–pituitary–adrenal axis responses to external stress.^100^ Recently, maternal programming effects have been reported to be involved in DNA methylation and histone modifications.^101^, ^102^ Not all tissues of a neonatal organ are equally exposed to environmental factors, and, the sensitivity to environment depends on the tissue type, thus, epigenetic alterations during postnatal stage are probably tissue specific.^64^ This would be interesting to investigate this stage further, since the majority of research on origins of diabetes so far have only considered pre‐gestational and gestational stage.

## 3 EPIGENETICS AND TRANSGENERATIONAL EFFECTS

**FIGURE 2:**
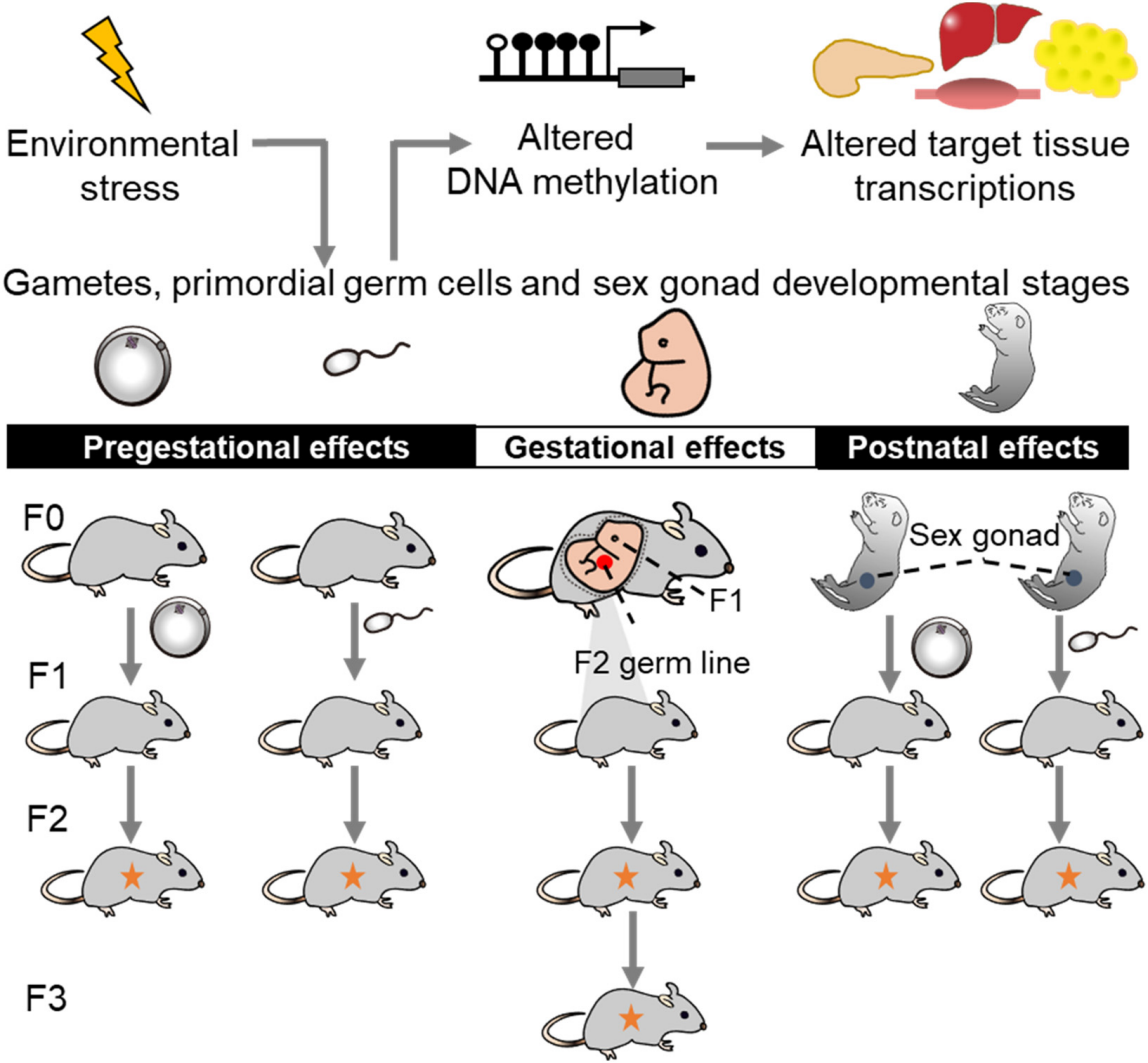
Transmission of epigenetically regulated transgenerational diabetes risk. Environmental factors exposure re‐programs the epigenome (DNA methylation) of the gametes before gestation, primordial germ cells during gestation, and sex gonad during postnatal period, which leads to alteration in DNA methylation status. These alterations are proposed to change the gene transcription in the main metabolic organs and tissues, promoting adult diabetes risk. Pre‐gestational effects. A founder female or male is exposed to environmental challenges in adult during gametogenesis, the gamete (oocytes and sperm) was also exposed. Both the F1 female and male were at risk of diabetes in adult. If these metabolic disorders transmit to the F2 offspring, the environmental factors will cause transgenerational effects. Gestational effects. A gestating female is exposed to abnormal environment. The F1 female and male were both at risk of diabetes in adult. The F2 offspring were at risk of diabetes as well, if the environmental derived traits are passed to the next generation via the gametes. True transgenerational effects appear when the acquired traits are transmitted to the F3 generation, because the germline of the F2 generation has not been previously exposed to the environmental triggers. Postnatal effects. A founder female or male is exposed to environmental challenges postnatally. Their germline (F1) is also environmentally exposed. If the metabolic traits passed to the next generation offspring (F2), the environmental triggers to F0 will cause true transgenerational effects.

There is interest in whether environmentally caused diabetic phenotype and epigenetic modifications can be inherited across generations (Table 1). The transgenerational inheritance is often described rather broadly to the phenomena that can be transmitted from one generation to the next. However, it is significant to distinguish intergenerational effects from transgenerational effects (Figure 2) that are found in generations who were not exposed to the initial environmental triggers. Epigenetic transgenerational effect means that the maternal or paternal individual already had the epigenetic alterations before gestation, and transmitted to F2 generation, while F1 offspring had no exposure to adverse environment during development. Although still largely unclear, transgenerationally inherited epigenetic markers could provide a new prospective to the alarming increase in diabetes incidence.

**TABLE 1:** Environmental factors induced the inheritance of diabetes risk and epigenetic alterations.

Representative paradigm	Species	Ontogenic stage	Inheritable pedigree	Phenotypic alterations	Target tissues or cell types	Epigenetic alteration in offspring target tissue genome	Epigenetic alteration in parental gametes genome	Refs
Pre‐gestational effects								
Maternal high fat diet	Mouse	Adult life	F3	Body size	Liver	Paternally imprinted loci were altered in F3	No reported	104
Maternal hyperglycemia	Mouse	Adult life	F1	Glucose intolerance	Pancreatic islets	Altered methylation and expression of *Gck* in F1	Changed methylation of *Gck* in F0 oocytes	75
Paternal pre‐diabetes	Mouse	Adult life	F2	Glucose intolerance	Pancreatic islets	More than 8000 regions differentially methylated in the pancreatic islets in offspring. And the altered DNA methylation of *Pik3ca*, *Pik3r1* and Ptpn1 in F1 and F2	*Pik3ca* and *Pik3r1* were altered DNA methylation in F0 sperm	67
Paternal high fat diet	Mouse	Adult life	F1	Glucose intolerance	Pancreatic islets	Altered metabolic gene expression in early embryos and islets in F1	Altered tsRNAs in F0 sperm	69
Gestational effects								
In utero undernutrition	Mouse	Adult life	F2	Metabolic disorder	Liver, brain	No alterations in methylation in F2 fetal liver and brain, but locus‐specific expression is perturbed	Over 100 loci exhibited differential patterns of DNA methylation in F1 sperm	107
n utero undernutrition	Mouse	Adult life	F2	Obesity and glucose intolerance	Liver	Altered DNA methylation and gene expression of the *Lxra* gene in F2	Modest alterations in DNA methylation of *Lxra* locus in F1 sperm	108
n utero undernutrition	Mouse	Adult life	F2	Obesity and glucose intolerance	Pancreatic islet	Not reported. Gene expression of *Abcc8* was altered in both F1 and F2	Not reported	109
In utero hyperglycemia	Mouse	Adult life	F2	Glucose intolerance	Pancreatic islet	An intragenic DMR of imprinting of the *Igf2/H19* locus in imprinting gene *Igf2/H19* was hyper‐methylated in both F1 and F2	Not reported. The *Igf2/H19* expression was decreased in F1 sperm	87
In utero hyperglycemia	Mouse	Adult life	F2	Obesity, insulin resistance and glucose intolerance	Liver, adipose and skeletal muscle	Altered DNA methylation and gene expression of the *Fry* in F1 and F2. No changes of *Fry* expression in F3	Altered methylome of D13.5 PGCs in male F1 offspring. DNA methylation status of target gene *Fry* was changed in F1 sperm, which was not found in F2 sperm	106
In utero high fat diet	Mouse	Adult life	F2	Glucose intolerance	Pancreatic islet	Not reported	Not reported	110
Postnatal effects								
High fat diet during lactation	Mouse	Adult life	F1	Obesity and glucose intolerance	Adipocyte	Not reported.	Not reported.	111
High‐carbohydrate during lactation	Mouse	Adult life	F1	Obesity and glucose intolerance	Skeletal muscle	*Slc2a4* promoter hypermethylation in F1 muscle	Not reported	97
Longer duration of breastfeeding	Human	17‐month‐old	F1	Not reported.	Blood cells	The promoter regions of *LEPTIN* hoper‐methylation modified in F1 blood samples	Not reported	98

For gamete origins of diabetes, the true transgenerational effects should be observed the diabetic phenotype and epigenetic changes in F2 offspring. As mentioned above, the example of paternal prediabetes induced diabetic inheritance showed the transgenerational effect in F2 generation. In their findings, paternal prediabetes altered methylome patterns in F1 sperm, and the effects of metabolic and epigenetic memory passed to the F2 generation.^67^ However, this transgenerational inheritance was not observed in maternal germline‐dependent diabetes inheritance. Latest study suggested that the effects of maternal hyperglycemia extend only to the F1 offspring and are unlikely to persist to the second generation.^75^

However, originating from prenatal stage, the diabetic susceptibility and epigenetic marks passed to the F3 generation are truly transgenerational effect, as the germline cells of F1 generation that produce the F2 offspring have been already affected during the embryonic development of the F1 generation. In rodent model, maternal HFD or diabetes during gestation has been reported to increase the risk of diabetes and obesity in both F1 and F2 generations.^103^ In a marked example, with female mice fed on HFD from 4 weeks from pre‐gestation until the end of lactation, the offspring displayed increased body length, mild insulin resistance and obesity in adulthood.,^103^, ^104^, ^108^ Interestingly, F3 female offspring generating from F2 males still showed an increase in body length and weight, in which true transgenerational effects have been detected.^104^ The phenomenon is suggestive of epigenetic inheritance, but the epigenetic modification was not reported.

Previous studies have now shown prominent effects of environmental toxicants exposure during gestation could pass on the influence to F3 generation, even F4 generation, through germline epigenetic alterations.^105^ Another view argued that postnatal disease phenotype may also influence the germ cells of the offspring themselves^67^, ^106^ which may cause the alteration in their gametes and the transmission effect. An animal study answered the question, in some part. They established a GDM mouse model and selected F1 male mice without the metabolic disorder phenotype to mate with healthy female mice to produce the F2 offspring. Then F2 male mice without the metabolic disorder phenotype were selected to mate with normal female mice to produce the F3 generation. They found that intrauterine exposure alone could lead to the epigenetic inheritance in F2 offspring but not F3 generation, and no methylation alteration of the targets genes was detected in F2 male germ cells and F3 somatic cells.^106^

## 4 CHALLENGES OF EPIGENOMICS STUDIES IN DEVELOPMENTAL ORIGINS OF DIABETES

Although researchers have gained advances with regard to understanding the epigenetic contribution to developmental origins of diabetes, there are still many challenges remain to be overcome. Epigenetic modifications do not change gene sequence, but cause the alterations of gene expression. Small methylation changes are reported as significant. Factually, in the existing animal and human research, only minor alterations at some loci were detected, and it is unknown whether it could cause a wider range of epigenetic effects as well. Additionally, such small alterations should be taken with caution because they might be caused by mixed cell types or altered cell‐type composition in target tissues.

Environmentally induced epigenome alterations often are minor, and variable among different individuals. Minor differences may be also due to stochastic changes. Also, manual technical variability could underlie a small difference reported in DNA methylation. High‐throughput sequencing approaches will further define the loci that are vulnerable to environmental insults. Thus, it seems too early to apply epigenetic modifications as biomarkers in public diabetes screening and drug target. Finally, it remains obscure what are the reasons that drive epigenetic changes. For instance, in the pre‐gestational diabetes model, it remains unknown what are the initiating signal or factor(s) that mediate the decreased Tet3 expression. These drivers that causes epigenetic changes may be the earlier target for diagnosis and intervention.

## 5 CONCLUSION

This review has summarized the evidence that the adverse nutritional, metabolic, or environmental chemicals exposure at important stages of early development could potentially alter the individual predisposition to diabetes and even have the transgenerational effect. There are accumulating data that demonstrate that an adverse environmental exposure is associated with a changed epigenetic landscape. At this point, identifying the epigenetically labile genes that are sensitive to environmental factors will probably find the potential target biomarkers for predicting diabetes risk. These novel biomarkers will excitingly have prospects of the early diagnosis and novel therapeutic approaches before diabetic symptoms develop. And if the combinations of the loci could be verified in large‐scale in human cohorts to monitor the effects, probably even across generations, which may revolutionize the diabetes medical care. Here is still a long way to explore, but undoubtedly, environmentally triggered disease risk provides a new perspective on diabetes.

## CONFLICT OF INTEREST STATEMENT

The authors declare that they have no conflict of interest.

## ETHICAL STATEMENT

Animal and human ethics are not involved in our present article.
